# Improving the Accuracy of Whole Genome Prediction for Complex Traits Using the Results of Genome Wide Association Studies

**DOI:** 10.1371/journal.pone.0093017

**Published:** 2014-03-24

**Authors:** Zhe Zhang, Ulrike Ober, Malena Erbe, Hao Zhang, Ning Gao, Jinlong He, Jiaqi Li, Henner Simianer

**Affiliations:** 1 National Engineering Research Center for Breeding Swine Industry, Guangdong Provincial Key Lab of Agro-Animal Genomics and Molecular Breeding, College of Animal Science, South China Agricultural University, Guangzhou, China; 2 Department for Animal Sciences, Animal Breeding and Genetics Group, Georg-August-Universität Göttingen, Göttingen, Germany; University of Miami, United States of America

## Abstract

Utilizing the whole genomic variation of complex traits to predict the yet-to-be observed phenotypes or unobserved genetic values via whole genome prediction (WGP) and to infer the underlying genetic architecture via genome wide association study (GWAS) is an interesting and fast developing area in the context of human disease studies as well as in animal and plant breeding. Though thousands of significant loci for several species were detected via GWAS in the past decade, they were not used directly to improve WGP due to lack of proper models. Here, we propose a generalized way of building trait-specific genomic relationship matrices which can exploit GWAS results in WGP via a best linear unbiased prediction (BLUP) model for which we suggest the name BLUP|GA. Results from two illustrative examples show that using already existing GWAS results from public databases in BLUP|GA improved the accuracy of WGP for two out of the three model traits in a dairy cattle data set, and for nine out of the 11 traits in a rice diversity data set, compared to the reference methods GBLUP and BayesB. While BLUP|GA outperforms BayesB, its required computing time is comparable to GBLUP. Further simulation results suggest that accounting for publicly available GWAS results is potentially more useful for WGP utilizing smaller data sets and/or traits of low heritability, depending on the genetic architecture of the trait under consideration. To our knowledge, this is the first study incorporating public GWAS results formally into the standard GBLUP model and we think that the BLUP|GA approach deserves further investigations in animal breeding, plant breeding as well as human genetics.

## Introduction

Predicting the yet-to-be observed phenotypes or unobserved genetic values for complex traits and inferring the underlying genetic architecture utilizing genomic data is an interesting and fast developing area in the context of human disease studies as well as in animal and plant breeding [1], [2], [3]. In this context, two predominant approaches were proposed: (i) whole genome prediction (WGP) [2], [4] and (ii) genome wide association studies (GWAS) [5], [6] or quantitative trait locus (QTL) mapping studies[7], [8], [9]. Both concepts use genomic and phenotypic data in a combined analysis.

GWAS take the road to detect markers significantly associated with a trait by setting a stringent *P*-value. Thousands of significant loci associated with complex traits have recently been found for model organisms [6], [10], [11], as well as crops [12], [13], [14], [15], [16], livestock [17], [18], [19], [20], [21] and the human population [22], [23], [24]. However, these loci typically explain only a small fraction of the total genetic variance. A prominent example is human height, for which tens of loci explain only ∼5% of the genetic variance [25], a phenomenon also called “missing heritability” in the literature [26], [27].

By fitting all markers in a prediction model simultaneously, whole genome prediction (WGP) has largely promoted the usage of whole genome markers, also revolutionizing commercial breeding systems and showing good results both in simulation studies [4], [28] and analyses of real data [29], [30], [31]. Furthermore, WGP is promising with respect to human disease studies [2], [32], [33]. The genetic architecture of the underlying complex trait together with the selected statistical prediction approach were found to have a large effect on the prediction accuracy [34], [35], [36]. Different prediction methods assume that the genetic effects of the loci follow a normal distribution [4], alternative distributions like the *t*-distribution [4], the double exponential distribution [37] or other distributions [38]. Performance of these models depends on how closely the model assumptions represent the true underlying genetic architecture [34], [35].

In the context of GWAS, it is not difficult to detect QTLs with large or moderate effects within large data sets for traits with high heritability [39], and it is also easier to conduct an accurate WGP in these cases [1]. However, the power to detect QTLs in a GWAS and the accuracy of WGP are unfavorable in case of small data sets and/or traits of low heritability [1], [39].

So far, results of GWAS and WGP have mostly been considered independently from each other, depending on whether the aim was to decode the genetic architecture (GWAS) or to accurately predict the unobserved phenotypes or genetic values (WGP). However, both approaches require the same type of data: a subset of a population for which phenotypes and genotypes are available. Since it is well known that the genetic architecture of complex traits affects the accuracy of genomic prediction [34], [35], [40], [41], some methods originally developed for WGP were recently used in a GWAS to detect loci significantly associated with the trait under consideration [20], [42]. Conversely, results from GWAS have already been pronounced to be useful to improve WGP [20]. However, it is yet to be investigated how to utilize significant QTLs identified in GWAS to improve WGP and to which extent existing knowledge of the genetic architecture of complex traits can help improving WGP.

In this study, we propose a new approach of utilizing already existing knowledge of genetic architectures in form of significant QTL regions obtained in independent association studies to improve the accuracy of WGP. This includes a new strategy of building trait-specific genomic relationship matrices used in a best linear unbiased prediction (BLUP) approach.

Besides the fact that the genetic architecture of a complex trait is known to affect the accuracy of genomic prediction as well as model selection [34], [35], there is another motivation for incorporating prior knowledge into the WGP model: WGP has always been performed within a specific population [4] or with the combination of raw data sets from several populations [31], [43], [44]. In these cases, the power of detecting and utilizing the genetic architecture is limited by the size of the data set used. In contrast to this, there is a large number of publicly available QTL regions and top SNPs detected in previous GWAS, which potentially reveal the genetic architecture of complex traits in a comprehensive way and which might therefore be used to enhance WGP in such a situation.

We will demonstrate in this study, that the performance of WGP can be improved by including the publicly available GWAS results (in case the genetic architecture is important for the complex trait under consideration) and that WGP accuracy can be improved especially in situations where the prediction accuracy is limited by a small sample size of the data set or a small heritability.

The remainder of the paper is organized as follows: We will first propose a generalized way of building genomic relationship matrices which are trait-specific. Based on this suggestion, we will illustrate with a dairy cattle and a rice data set that using already existing GWAS results from publicly available databases to build trait-specific genomic relationship matrices improves the accuracy of WGP compared to two well investigated WGP approaches: GBLUP [45] and BayesB [4]. We will finally study the impact of sample size and heritability on the relative performance of our approach with simulated data and discuss the implications of the new approach, which we term “BLUP|GA” (“BLUP approach given the Genetic Architecture”) in the following.

To our knowledge, this is the first study proposing a formal way to improve the accuracy of WGP by directly incorporating results from publicly available GWAS results and which validates the effectiveness of the new approach using real data sets.

### Methodology: A New Approach for Building Trait-Specific Genetic Variance-Covariance Matrices

Several approaches have already been proposed for building genomic relationship matrices by estimating the realized genomic relationship matrix [45], [46], [47], [48]. And various rules were tested to correct the genotype matrix for allele frequency at single marker level to centered and standardized marker genotypes [45], [46], [48]. All of these rules aim at obtaining an unbiased estimate of the relationship coefficient between pairs of individuals, and all of them assume that the effects of all loci are drawn from the same normal distribution.

Following the approach of VanRaden [45], the commonly used genomic relationship matrix **G** is defined as



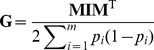
(1)


Here, **I** is an identity matrix and the matrix **M** contains the corrected SNP genotypes, with the number of rows equal to the number of individuals and the number of columns equal to the number of markers. Genotypes are coded as 0, 1 and 2, representing the number of copies of the second allele. For locus *i*, the original genotype is corrected for the allele frequency of the second allele at locus *i* in the base population by subtracting 2*p_i_*. We used a uniform value of *p_i_* = 0.5 for all SNPs to build the genomic relationship matrix in this study, since the accuracy of WGP is known to be unaffected by the use of different allele frequencies for correction [48], [49], [50]. By using the identity matrix **I** in equation (1), it is implicitly assumed that all loci contribute equally to the variance-covariance structure.

In general, the variance contribution for different loci may be different [51], since the distribution of effect sizes is variable across traits. Zhang *et al.* [51] therefore proposed to use a trait-specific matrix **TA**, given by



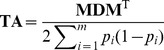
(2)where **D** is a diagonal matrix with marker weights for each locus on the diagonal to represent the relative size of variance explained by the corresponding loci. In the present study, we propose to use a similar approach, in which only a subset of “important” markers are weighted accordingly, instead of assigning variable weights to the full set of available markers. This approach is computationally less demanding when building the covariance matrix. Since for most quantitative traits only a very small proportion of loci was found to have significant effects and a large number of other loci was found to have very small effects (see e.g. adult height in humans [25], [52] or flowering time in maize [13]), a realistic weighting strategy is giving individual and large weights to loci with large effects, and relatively smaller and uniform weights to the rest of the loci. Based on this, we can divide the *m* available markers into two groups, including *m*_1_ markers with large and 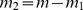 with small effects. In the following, the marker genotype matrices for these two marker groups will be denoted by **M**_1_, and **M**_2_, including *m*_1_ and *m*_2_ markers, respectively, and **M** will be sorted such that **M** = [**M**_1_, **M**_2_]. In this study, classification of the markers to **M**_1_ was based on GWAS results obtained from public database, and this is described in the section ‘Approach to infer marker weights from GWAS results’.

We will further use an overall weight ω for large effect markers in **M**_1_ and we define 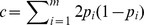 as well as 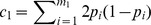.

We finally propose to use the matrix
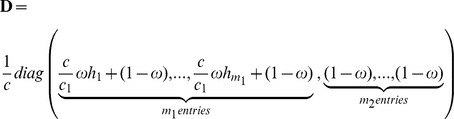
in equation (2), where *h*_1_, *h*_2_, …,*h_m_*_1_ are certain marker weights which have to be obtained beforehand. This approach is equivalent to using
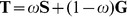
(3)


with 
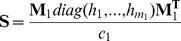
 and 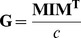
as new trait-specific variance-covariance matrix. Hereby, **S** is based on the set of markers being “important” for the considered trait, whereas **G** corresponds to the standard genomic relationship matrix proposed by VanRaden [45]. Note that when we use equal allele frequencies (*p_i_* = 0.5) in *c* and *c*_1_, then
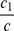
is the proportion of all markers which are contained in **M**_1, that is_ 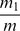. The matrix **S** is supposed to capture the genetic architecture part for the trait under consideration. Further note that **T** equals **G** for 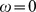
_,_ and that it equals **TA** [51] with **D** = 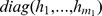
in case 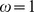
and 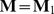.

To build the **T** matrix given in equation (3), three additional parameters are needed: the subset of *m*_1_ markers to build **S,** the overall weight ω for **S**, and a vector of marker weights 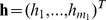 corresponding to each marker used in **S**. Note that in the present study the vector of weights **h** was always rescaled after choosing its components by multiplying each entry by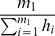 to keep the S and G being in the same scale.

In the following, we will consider these three parameters as variables which have to be specified within a study. The subset of *m*_1_ markers and their corresponding weights can thereby be chosen very flexible, for example as (i) estimated marker effects or variances for a proportion of top markers from genomic prediction; (ii) estimated effects or variances for markers in the QTL regions detected by GWAS; or (iii) counts of how often a marker was reported to belong to a (significant) QTL region in the literature, thus allowing to incorporate prior knowledge of the underlying genetic architecture of the complex trait under consideration.

We finally propose to use **T** (instead of **G** or **TA**) as variance-covariance matrix in a genomic best linear unbiased prediction (BLUP) model. We will call this approach BLUP|GA (“BLUP approach conditional on the Genetic Architecture”).

## Results

In the following, we will present WGP results for a real dairy cattle and a rice data set using the methodology introduced above. Predictive ability of the WGP was measured via different cross-validation procedures, applying the BLUP|GA approach with genetic covariance structure given by the trait-specific variance-covariance matrix **T** as proposed in equation (3). The weights **h** in **T** were chosen based on counts of how often a marker was reported to be within a significant QTL region during association studies previously carried out in the literature, a knowledge we will retrieve from publicly available QTL databases. We will compare the performance of BLUP|GA with the standard GBLUP approach [45] and with BayesB [4]. Further details can be found in the ‘Material and Methods’ section.

### Dairy Cattle Data

We considered 2,000 bulls of the German Holstein population which were genotyped with the Illumina Bovine SNP50 Beadchip. After quality control 45,221 autosomal SNPs were used in the study. We analyzed the traits milk fat percentage (FP), milk yield (MY) and somatic cell score (SCS) and used accurately estimated breeding values (EBVs) from the conventional breeding value estimation as quasi-phenotypes in the whole genome prediction models (Table 1).

**Table 1:** Summary statistics of data sets and corresponding traits.

Data set	Trait	N	Mean^a^	S.D. a	r^2^/h^2^ b
Cattle	Fat percentage	2000	−0.027	0.294	0.973
	Milk yield	2000	231.7	649.8	0.973
	Somatic cell score	2000	103.1	11.6	0.942
Rice	Days to flower (Arkansas)	374	87.94	12.63	0.785
	Flag leaf length	377	30.63	5.74	0.763
	Flag leaf width	377	1.22	0.25	0.717
	Panicle number per plant	372	3.25	0.41	0.646
	Plant height	383	116.60	21.09	0.832
	Panicle length	375	24.37	3.54	0.781
	Primary panicle branch number	375	9.94	1.78	0.621
	Seed number per panicle	376	4.85	0.33	0.678
	Seed Width	377	3.12	0.39	0.924
	Blast resistance	385	5.04	2.94	0.762
	Amylose content	401	19.88	5.46	0.900

a mean and standard deviation (S.D.) of conventional estimated breeding values for cattle traits or phenotypes for rice traits;

b reliability (*r* ^2^) for cattle trait EBV, or heritability (*h* ^2^) for rice trait phenotypes.

Marker weights for the BLUP|GA approach were obtained by using publicly available GWAS results stored in the database animalQTLdb [17] and based on the number of publications reporting a significant QTL region including the corresponding marker. Details on this are given in the ‘Material and Methods’ section. We performed 20 replicates of a five-fold cross-validation to obtain an average predictive ability for BLUP|GA, GBLUP, TABLUP and BayesB for three different population sizes.

**Figure 1:**
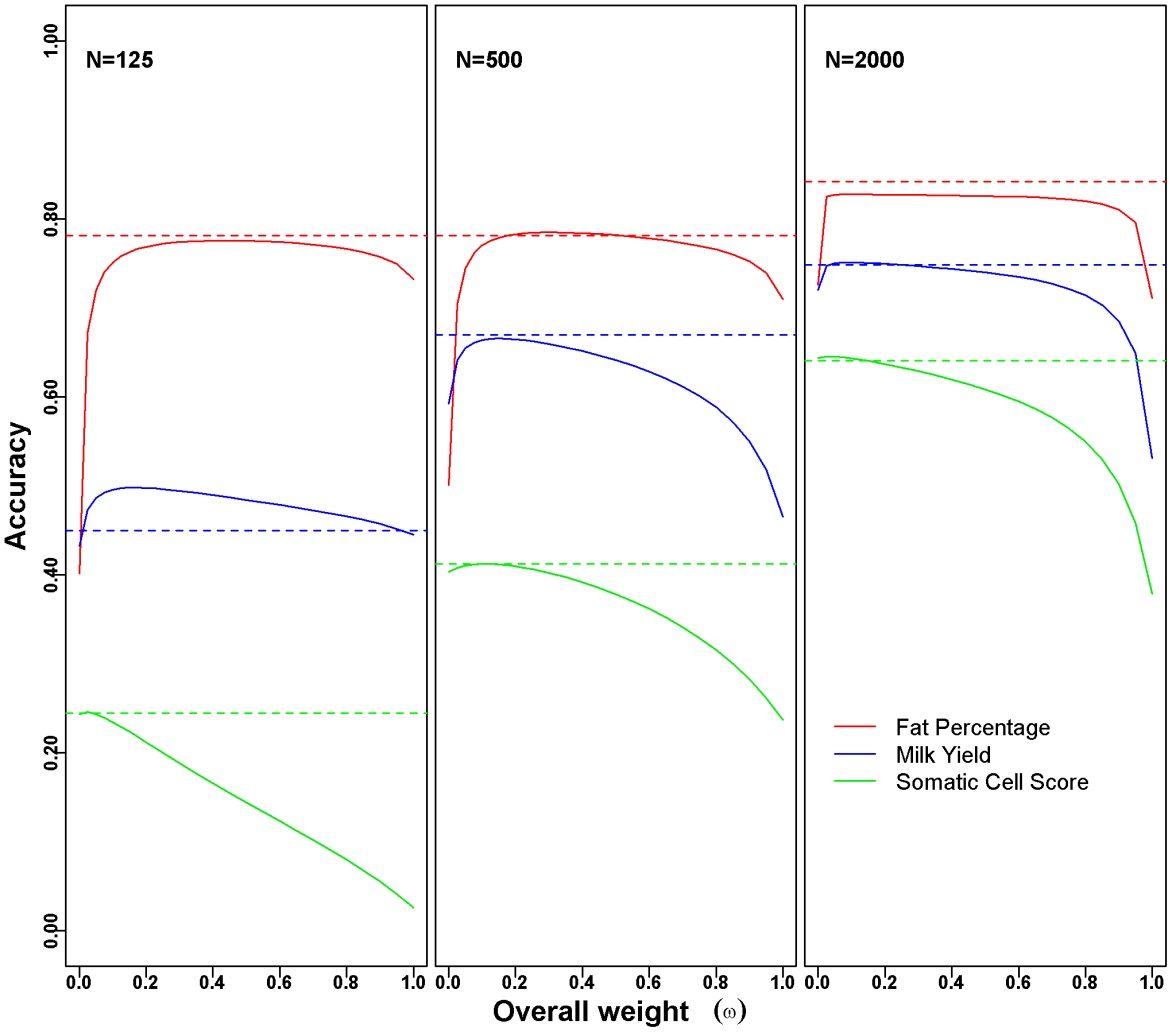
Accuracies of WGP in dairy cattle data set. The solid lines show the change of BLUP|GA accuracy with the overall weight (ω) for fat percentage (red), milk yield (blue), and somatic cell score (green). SNP weights in the BLUP|GA approach were based on the number of QTL reports as described in the ‘Material and Methods’ section. GBLUP corresponds to the scenarios with overall weight ω = 0, and the accuracies of BayesB are presented by horizontal colored dash lines. Accuracies were calculated as the mean of 20 replicates of five-fold cross-validation with variable population size (*N* = 125, 500 and 2000).

Results in terms of accuracies are reported in Table 2 and Figure 1. The BLUP|GA method outperformed the standard GBLUP approach for all three model traits in terms of accuracy (Table 2). This could be observed for all three population sizes. The superiority of BLUP|GA increased with the extremity of the underlying genetic architecture of the complex trait. This characteristic is similar to that of BayesB, which is also favorable for traits affected by large-effect QTLs [34], [35]. Since the **T** matrix used in the BLUP|GA model is a mixture of the **G** matrix and the **S** matrix, we had to choose an overall weight ω for the **S** matrix. The accuracies of BLUP|GA increased for FP and MY when increasing ω from 0 to 1, with a drop in accuracy for ω approaching 1 (Figure 1). For SCS, the accuracy decreased continuously with increasing ω. Note that accuracies reported for BLUP|GA in Table 2 correspond to the overall weight ω which led to the highest average accuracy. The BLUP|GA approach requires far less computing time as BayesB although it enables a differentiated treatment of the SNPs (Figure S1).

**Table 2:** Accuracy and unbiasedness of WGP for dairy cattle.

		Fat percentage		Milk yield		Somatic cell score	
*N*	Method	*r*_(EBV, GEBV)_	*b*_(EBV,GEBV)_	*r*_(EBV, GEBV)_	*b*_(EBV,GEBV)_	*r*_(EBV, GEBV)_	*b*_(EBV,GEBV)_
2000	BLUP|GA	0.824±0.001	0.975±0.001	**0.751**±0.001	**1.025**±0.002	**0.646**±0.001	1.029±0.002
	BayesB	**0.842**±0.000	**0.985**±0.001	0.749±0.001	1.027±0.002	0.641±0.001	1.069±0.003
	GBLUP	0.726±0.001	1.028±0.002	0.720±0.001	1.042±0.002	0.644±0.001	**1.026**±0.002
	TABLUP	0.806±0.001	1.021±0.001	0.738±0.001	0.973±0.002	**0.646±0.001**	0.932±0.002
500	BLUP|GA	**0.785**±0.001	1.017±0.003	**0.673**±0.003	**1.133**±0.005	**0.412**±0.004	0.952±0.011
	BayesB	0.781±0.001	**1.007**±0.002	0.670±0.002	1.137±0.006	0.398±0.005	1.075±0.014
	GBLUP	0.501±0.003	0.986±0.007	0.593±0.004	1.169±0.007	0.403±0.005	**0.954**±0.012
	TABLUP	0.684±0.002	1.174±0.004	0.626±0.004	1.115±0.007	0.405±0.005	0.856±0.010
125	BLUP|GA	0.776±0.003	**1.042**±0.008	**0.506**±0.007	**1.203**±0.020	0.245±0.013	0.954±0.057
	BayesB	**0.782**±0.004	1.109±0.010	0.450±0.009	1.774±0.039	**0.248**±0.012	1.124±0.060
	GBLUP	0.401±0.011	1.214±0.035	0.432±0.010	1.379±0.034	0.243±0.013	**0.966**±0.058
	TABLUP	0.532±0.009	1.443±0.026	0.434±0.009	1.233±0.032	0.246±0.012	0.831±0.041

Mean (± standard error of means) of accuracy (*r*) and unbiasedness (*b*) were calculated from 20 replicates of five-fold cross-validation for each of the three traits. The best result in each block is printed in boldface.

**Figure 2:**
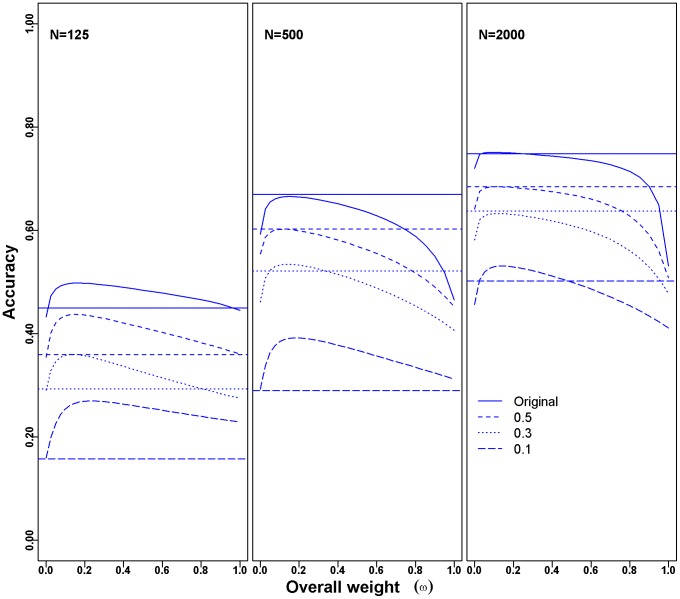
Accuracy of WGP for simulated traits with different heritabilities and sample sizes. The curves show the change of the accuracy obtained with BLUP|GA for varying overall weight ω for milk yield. Different curves represent the accuracies obtained from traits with original phenotype (solid line), or simulated phenotypes with heritability of 0.5 (short-dashed line), 0.3 (point line) and 0.1 (long-dashed line), respectively. The numbers of QTL counts were used to infer the marker weights for the BLUP|GA approach. The GBLUP approach corresponds to scenarios in which ω = 0 (starting points for each curve), and the accuracies of BayesB are presented by horizontal lines. Accuracies were calculated as the mean of 20 replicates of five-fold cross-validation with different population sizes (N =  125, 500, and 2000).

To investigate the performance of WGP in more challenging situations, we simulated traits with lower heritability based on the original MY breeding values. For each of the three population sizes, a random error was added to the original phenotypes (EBV of MY) to generate a “new” trait with lower heritability. The average accuracies of BLUP|GA and BayesB for 20 replicates of five-fold cross-validation for the original phenotypes as well as the artificial low heritability traits are shown in Figure 2. The accuracy decreased with the population size and trait heritability (as expected) for all three approaches. Additionally, it could be observed that the accuracy of BLUP|GA was higher than that of GBLUP in all considered scenarios (Figure 2). BLUP|GA showed no advantage over BayesB for the original phenotype with high heritability, but outperformed BayesB when the population size was small or when the trait heritability was low (Figure 2). The corresponding average values of accuracy and unbiasedness for GBLUP, BayesB and the best scenario (“best” with respect to the optimal value of ω, and the optimal subset of SNP listed in Table 3) for BLUP|GA are presented in Table S1.

**Table 3:** SNP list summary.

Data set	Trait	Total QTL^a^	Number of SNPs with QTL count^b^> =				
			1	2	3	5	10
Cattle	Fat percentage	279	1325	257	135	57	1
	Milk yield	247	1622	250	107	85	0
	Somatic cell score	169	993	184	66	1	0
Rice	Days to flower (Arkansas)	38	6488	2196	292	204	0
	Flag leaf length	110	13652	3467	1551	1019	0
	Flag leaf width	106	13689	7658	3237	560	58
	Panicle number per plant	197	19968	11031	6605	3765	672
	Plant height	979	34240	31030	26430	14029	6791
	Panicle length	240	23942	16521	10865	4164	477
	Primary panicle branch number	52	7207	2769	465	0	0
	Seed number per panicle	58	17487	9722	1424	48	0
	Seed Width	31	4998	840	88	0	0
	Blast resistance	169	18628	13194	6631	2076	190
	Amylose content	50	6059	1916	1037	165	0

a Total QTL: Total number of QTL regions for each trait obtained from animalQTLdb (Release 18) [17] and Gramene (Release 36) [16].

b QTL Counts were obtained as described in the ‘Material and Methods’ section. The exact number of top SNPs used in final analysis were showed in bold face.

### The Rice Diversity Panel

We used 413 inbred accessions of *Oryza sativa* from the Rice Diversity Panel data set (cf. Zhao *et al.* [53]), which were genotyped for approximately 37,000 SNPs; 11 different traits were considered in our analyses (Table 1). Marker weights for the BLUP|GA approach were obtained using GWAS results stored in the Gramene database [16]. More information is given in the ‘Material and Methods’ section.

We found that BLUP|GA yielded the highest average accuracy across all the 11 traits (Table 4). It outperformed GBLUP for nine out of the 11 traits, either in terms of accuracy or in terms of unbiasedness. On average, BLUP|GA showed an advantage over GBLUP and BayesB by 0.01 in accuracy, while GBLUP and TABLUP performed equally well (Table 4). BayesB performed slightly better than BLUP|GA for two out of the 11 traits, and worse than GBLUP on five traits. Compared to GBLUP, BLUP|GA had the highest increase in accuracy for the traits “days to flower” (0.036, 5.4%), “amylase content” (0.020, 2.5%), and “blast resistance” (0.014, 2.0%), which indicates that the existing knowledge on the genetic architectures underlying these traits can indeed enhance WGP. The BLUP|GA approach improved the unbiasedness of prediction for nine out of the 11 traits compared to GBLUP (Table 4).

**Table 4:** Accuracy and unbiasedness of WGP for rice.

Trait	*r*_(PHE, GEBV)_				*b*_(PHE,GEBV)_			
	BLUP|GA	BayesB	GBLUP	TABLUP	BLUP|GA	BayesB	GBLUP	TABLUP
Days to flower (Arkansas)	**0.700**±0.003	0.675±0.011	0.664±0.003	0.663±0.003	**1.001**±0.004	1.013±0.010	1.051±0.005	0.952±0.006
Flag leaf length	**0.516**±0.003	0.513±0.003	0.505±0.003	0.514±0.003	0.942±0.008	0.970±0.009	**0.999**±0.008	0.860±0.007
Flag leaf width	**0.766**±0.002	0.765±0.002	0.757±0.002	0.759±0.002	1.041±0.003	**1.029**±0.003	1.057±0.003	0.984±0.003
Panicle number per plant	**0.822**±0.001	0.820±0.001	0.821±0.001	0.814±0.002	1.016±0.002	**1.014**±0.002	1.021±0.002	0.976±0.003
Plant height	**0.760**±0.002	0.751±0.002	0.753±0.002	0.753±0.002	1.061±0.003	**1.043**±0.003	1.056±0.003	1.011±0.003
Panicle length	**0.661**±0.004	0.657±0.004	0.659±0.004	0.651±0.004	**0.994**±0.006	0.984±0.007	0.991±0.006	0.905±0.006
Primary panicle branch number	**0.626**±0.003	0.625±0.003	0.625±0.003	0.625±0.003	**1.024**±0.006	1.030±0.006	1.044±0.006	0.913±0.006
Seed number per panicle	**0.579**±0.004	0.572±0.004	0.575±0.004	0.568±0.004	1.121±0.007	**1.053**±0.006	1.118±0.007	0.914±0.005
Seed Width	0.837±0.001	0.842±0.005	0.837±0.001	**0.852**±0.001	1.027±0.003	0.967±0.008	1.026±0.003	**1.015**±0.003
Blast resistance	0.703±0.003	**0.704**±0.003	0.689±0.003	0.690±0.003	1.043±0.004	1.031±0.005	**0.998**±0.005	0.964±0.005
Amylose content	**0.825**±0.004	0.801±0.005	0.805±0.005	0.805±0.005	**1.013**±0.004	0.934±0.010	1.031±0.006	0.980±0.005
AVERAGE	**0.709**	0.702	0.699	0.699	1.026	**1.006**	1.036	0.952

Mean (± standard error of means) of accuracy (*r*) and unbiasedness (*b*) were calculated from 20 replicates of five-fold cross-validation for each trait. The best result in each block is printed in boldface. Average accuracy (*r*) and unbiasedness (*b*) were calculated for each method across all 11 traits.

BayesB outperformed BLUP|GA only for “seed width” and “blast resistance” (Table 4). This suggests that the existing knowledge from the QTL list [54] for these two traits is not more promising than the one extracted from the rice diversity panel itself. To validate this assumption, we ran BLUP|GA using an **S** matrix build from the top SNPs selected by the size of estimated marker effects from the equivalent model of GBLUP [55] obtained within each fold of the 20 replicates of five-fold cross-validation. The average accuracies of BLUP|GA from this scenario were 0.861 (±0.001) and 0.704 (±0.003) for “seed width” and “blast resistance”, respectively. The increased accuracy in this additional scenario and the small number of known QTL (31, Table 3) for “seed width” suggest that the underlying genetic architecture for this trait within the rice diversity panel might be different from that obtained from the GWAS list and that the QTL list might be too short to reflect the complete genetic architecture for this trait. The TABLUP result (0.852, Table 4) also confirmed our assumption.

## Discussion

We proposed a new WGP approach called BLUP|GA. One plausible feature of BLUP|GA is the fact that any existing knowledge of the genetic architecture of the complex trait under consideration can be fitted into this prediction model by choosing the corresponding marker weights in equation (3), which can potentially improve the predictive ability of WGP. In this study, we used publicly available QTL lists as the prior knowledge of the underlying genetic architecture (“GA”) in an application of a dairy cattle and a rice data set. Results indicated that the publicly available QTLs identified from hundreds of association studies can help to improve the accuracies of WGP via the BLUP|GA model and that the BLUP|GA approach dominates two influential WGP methods, GBLUP and BayesB, for the data sets considered in this study. The BLUP|GA approach therefore provides a flexible connection between WGP and the existing knowledge of the genetic architecture of complex traits as given by association studies.

### BLUP|GA incorporates prior knowledge of the underlying genetic architecture

**Figure 3:**
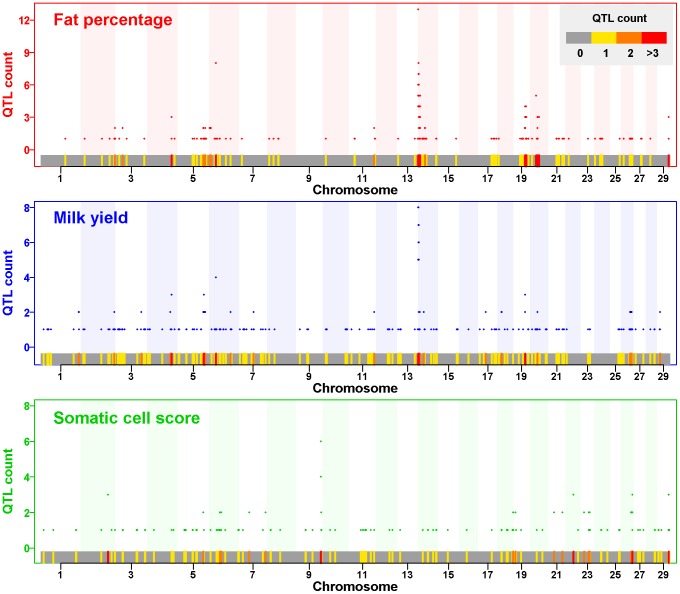
Distribution of reported QTLs positions and marker weights obtained from QTL list. Reported QTLs associated with fat percentage (red), milk yield (blue) and somatic cell score (green) retrieved from animalQTLdb (http://www.animalgenome.org/animlQTLdb) [17]. Marker weights were calculated as the number of times that each marker was reported to be within a significant QTL region (QTL counts). The colored bar under each plot shows the distribution of QTL positions across the whole genome for the three traits with color keys defined in the first plot (top-right).

The most important difference between BLUP|GA and any other WGP approach is that BLUP|GA can enhance the accuracy of WGP by modeling *any* “existing knowledge” of the GA, including publicly available GWAS results. This can be achieved in three steps: (i) building the **S** matrix based on a list of important markers and their corresponding weights which are obtained from “existing knowledge”, (ii) forming the **T** matrix as the weighted sum of **S** and **G** using equation (3), and (iii) predicting the genetic merit of all individuals by solving the mixed model equations, in which the covariance structure is given by the **T** matrix. SNPs used to build **S** should lie in trait associated chromosomal regions and their corresponding marker weights should represent their relative contributions. In this study, we obtained the list of important SNPs and their corresponding marker weights for different traits within a dairy cattle and a rice data set from QTL databases which are publicly available (Table 3, Figure 3).

We showed that GWAS results are not only useful for follow-up studies in the context of association studies, but also for WGP. For two out of the three dairy cattle model traits, the accuracies of the BLUP|GA approach showed an “n” type curve (Figure 1), which suggests that neither the **G** matrix (ω = 0) nor the **S** matrix (ω = 1) alone, but rather the **T** matrix as a mixture of both, is the most appropriate variance-covariance matrix with respect to the predictive ability in the standard GBLUP approach.

Our study also gives an answer to the question raised by human genetics “to what extent GWAS have identified genetic variants likely to be of clinical or public health importance” [23]. Our results show that GWAS results are useful for the prediction of genetic merits in animal and plant breeding, and this might also be valid for the prediction of disease risk in humans and therefore deserves more exploration in the future.

### Computational efficiency

With the fast increase of the data volume available, the computational efficiency of a whole genome approach becomes a critical issue in the post-genomic era. The BLUP|GA approach shares similar computational characteristics with the GBLUP approach, which is time and memory efficient, especially when the **G** matrix has been built and stored before running a job (Figure S1). On the contrary, Bayesian modeling is computationally intensive, and it usually takes hours to run analyses of data sets based on high density SNP chips (Figure S1, [33]), and days to run analyses of data sets based on whole genome sequences [56]. With the decrease of sequencing costs, the p>n problem will become even more serious for WGP approaches. The relationship matrix based approach gains attractiveness in this situation, since it can manage the same prediction problem in the dimension of number of individuals rather than the number of markers.

### QTL lists from GWAS results

Our results demonstrated that the comprehensive QTL list collected from GWAS and QTL mapping studies can be used to improve the performance of WGP via the BLUP|GA model. In the past decade, the genetics community conducted thousands of phenotype-genotype association studies to dissect the genetic architecture of complex traits in animals [17], [18], [19], [20], [57], plants [12], [13], [14], [15] and humans [22], [23], [58]. Finally, hundreds of QTLs were detected to be associated with each of the traits of interest, such as MY in dairy cattle (Table 3) [17], plant height in rice (Table 3) [54] or adult human height [52], [59]. One usual strategy to utilize these results is to sift out most promising SNPs for follow-up replication studies to determine true association findings in previous GWAS [60], [61], although it usually takes years or longer from a QTL to a validated gene [62]. Alternatively, our results have shown that utilizing the QTL list via the BLUP|GA approach, one can benefit from more accurate GEBVs in animal and plant breeding programs or from more accurate predictions of individual genetic risk of complex disease in humans, although the exact functions and relationships of all genes underlying the complex trait under consideration are not known yet.

The QTL list used for BLUP|GA may come from hundreds of studies and hence is the most comprehensive profile of the underlying genetic architecture that is available. This is evidenced by the similar shape of profiles obtained by our analyses of the cattle QTL list (Figure 3) and the estimated marker effects for MY in the cattle data set (Figure S2). By counting the significant QTLs and inferring the corresponding weights for each marker for a trait, we can account for relatively more important regions across the whole genome, which is the kind of model selection we are interested in.

### Genetic architecture and accuracy of WGP

The genetic architecture of a complex trait is one of the most influential factors for WGP [34], [35]. Generally, if a trait is controlled by only a few major genes, methods with an explicit model selection are known to work best in WGP and these major genes should easily be detected in a GWAS. In case no major genes exist, it is hard to detect moderate or small effect QTLs in GWAS [25], [63], and the GBLUP method usually performs better.

From a WGP perspective, our results for the three model traits in the dairy cattle data set (Figure 1, Table 2), as well as several studies using simulations [34], [41] or real data [35], [40], have clearly confirmed this hypothesis. Considering the dairy cattle data set, using the BLUP|GA method improved the accuracy of WGP for traits with a characteristic genetic architecture, such as FP and MY, but not for a trait without evidence of a characteristic trait genetic architecture, such as SCS. For the rice traits, more significant QTL regions were identified for the plant height than panicle length (Table 3, Figure S3), and we obtained more gain in accuracy for plant height (Table 4). It would be interesting to explore the performance of the BLUP|GA approach with other species as well. This is left for future work.

As the effect size of detectable QTL decreases with the increase of population size, a training population with sufficient size (*N*_s_), suitable population structure and accurate phenotypes is usually needed to detect the genetic architecture of a complex trait [39]. The required sample size *N*_s_ to achieve a certain accuracy will be different for different species and populations according to their effective population size (*N*_e_) and genome length [34], [47]. In this study, the Germany Holstein dairy cattle population was taken as an example (*N*_e_ = ∼100 [64]), and the training population sizes used in the study were approximately 1 *N*_e_ (100), 4 *N*_e_ (400) and 16 *N*_e_ (1,600), respectively. These training population sizes are large relative to the small value of *N*_e_ (compared to other common species such as humans (*∼*10,000) [65], [66], mice (>20,000)[67] and swine (∼100 for one breed) [68], [69]. The decreased accuracies (Figure 1, Figure 2) and the shrunk estimated marker effects (Figure S2) indicate that the power of detecting genetic architecture and the predictive ability of WGP is seriously affected by the training population size as well as the accuracy of phenotype (heritability). Incorporating existing knowledge of the underlying genetic architecture into the WGP model (such as QTL lists from previous publications) therefore appears to be even more reasonable when the population size is small and the heritability is low. The new approach is more potent in case the combining of raw data sets are less possible, which was confirmed by our simulation results from the cattle data set (Figure 2, Table S1).

The new approach presented in this study still offers room for further improvements, such as refining the SNP list and marker weights obtained from QTL lists or modifying the **T** matrix while combining the information from **G** and **S**. We have tried to base weights in **h** on accumulated *P*-values rather than the number of citations, which basically led to very similar findings (results not shown). Other concepts like including e.g. pathway information might be promising as well and are left for further studies.

## Conclusions

The BLUP|GA method provides a new tool to incorporate existing knowledge of the genetic architecture of complex traits explicitly into a genomic prediction model. Using the BLUP|GA model, we illustrated that the publicly available QTL lists detected by hundreds of GWAS and QTL mapping studies improved the performance of WGP compared to standard WGP methods within a dairy cattle and a rice data set, respectively. The accuracy of WGP could be improved for two out of the three model traits in dairy cattle and for nine out of 11 traits in the rice diversity panel. The publicly available GWAS results were shown to be potentially more useful for WGP utilizing smaller data sets and/or traits of low heritability, depending on the genetic architecture of the trait under consideration. BLUP|GA also improved the prediction accuracies compared to the traditional methods GBLUP and BayesB. To our knowledge, this is the first study incorporating public GWAS results into the standard BLUP model and we think that the BLUP|GA approach deserves further investigations in animal breeding, plant breeding as well as human genetics.

## Materials and Methods

A dairy cattle and a rice data set were analyzed in this study. Summary statistics for these sets and the considered traits are given in Table 1.

### The German Holstein Population

Genotypic data from the Illumina Bovine SNP50 Beadchip [70] was available for 5,024 German Holstein bulls. SNPs with a minor allele frequency lower than 1%, with missing position or a call rate lower than 95% were excluded. After filtering, there were 42,551 SNPs remaining for further analyses. Imputation of missing genotypes at these SNP positions was done using Beagle 3.2 [71]. For all bulls, conventional estimated breeding values for milk fat percentage (FP), milk yield (MY) and somatic cell score (SCS) with reliabilities greater than 70% were available.

The three traits, FP, MY and SCS, were considered due to their well-established distinct genetic architectures. For FP, a single mutation in the diacylglycerol acyltransferase 1 (*DGAT*1) gene explains approximately 30% of the genetic variance in Holstein Friesian cattle [60], [72]. For MY, several moderate effect loci have been detected, whereas for SCS, which is a health index counting the number of somatic cells in milk, only loci with small effects have been reported so far, so that it can be considered as a trait exhibiting a quasi-infinitesimal mode of inheritance. These three traits therefore represent three different possible genetic architectures of complex traits.

For our further studies, we chose to use the 2,000 bulls with the highest reliabilities in the trait MY to decrease the time demanding. In order to consider two additional scenarios with even smaller population size, we randomly selected a subset of 500 and 125 individuals out of these 2,000 individuals. To investigate the effect of different heritabilities, we also created new phenotypes for the bulls by adding random error terms to the conventional estimated breeding values such that the heritability of the new phenotypes was 0.5, 0.3 and 0.1, respectively.

### The Rice Diversity Panel

The rice diversity panel consists of 413 inbred accessions of *Oryza sativa* collected from 82 countries [53]. They were systematically phenotyped for 34 traits and genotyped with a custom-designed 44,100 oligonucleotide genotyping array. In total, we used 36,901 SNPs in the present study. We considered a subset of 11 (listed in Table 1) out of the 34 traits, which have more than 30 QTL reports respectively. Phenotypes and genotypes are publicly available from http://www.nature.com/ncomms/journal/v2/n9/full/ncomms1467.html [53] and http://www.ricediversity.org/data/sets/44 kgwas/. For more details about the rice diversity panel we refer to Zhao *et al.* [53].

### Approach to infer marker weights from GWAS results

For a given trait of interest, we first extracted a full list including the “most important SNPs” with respect to this trait, for which the according weights have to be chosen in a second step. These are the SNPs which are finally used to build the **S** matrix in the BLUP|GA approach.

We first retrieved regions of QTLs associated with the trait under consideration from the literature. For each reported QTL, we picked the SNPs from the genotype data set located in the corresponding QTL region. If a reported QTL region did not contain any SNP, we extended the QTL region by 300 kb at both sides to track the SNPs nearby. If a reported QTL region contained more than 1,000 SNPs, the corresponding QTL report was excluded from our analysis, since this QTL would not be informative with respect to the marker weights obtained in the next step. We thereby obtain a list of the most important SNPs as well as a list of corresponding QTL regions. For each SNP in this list, we then calculated its marker weight for the trait specific matrix **S** used in the BLUP|GA approach by counting the number of publications which report a significant QTL region which is included in the QTL list and which contains the considered SNP. Finally, we removed a marker from the SNP list, if its corresponding QTL count did not exceed 1 in order to minimize the effect of potential false positive QTL(s) to the marker weights.

### Marker weights for the dairy cattle data set

A list of significant QTLs for the dairy cattle data set was obtained from animalQTLdb [17] (http://www.animalgenome.org/QTLdb, Release 18, October 2, 2012), which is a comprehensive QTL database for domestic animals. This list included 5,920 QTLs on 407 traits from 331 publications. For each QTL, the estimated QTL intervals in base-pairs (bp), the associated trait, the significant *P*-value and other related information were given. For more details, we refer to Hu [17] and http://www.animalgenome.org/QTLdb. There were 279, 247 and 169 QTLs reported for FP, MY and SCS, respectively (cf. Table 3). Applying the approach described above to obtain a list of QTL regions, 194, 210 and 124 QTLs were finally included in our further analyses. The number of SNPs from the genotype data which were located in these QTL regions and the number of QTL reports for these SNPs are summarized in Table 3. The reported QTLs for FP are clustered on chromosomes 6, 14 and 20, while the positional distributions for QTLs associated with SCS trend to be evenly spaced across the whole genome (Figure 3). The final marker weights (QTL counts, obtained by the procedure described in the previous section) are also plotted in Figure 3. The annotation information for the SNPs and the corresponding marker weights are provided in Table S2.

### Marker weights for rice data set

The QTL list for *Oryza sativa* (rice) was obtained from the Gramene database (ftp://ftp.gramene.org/pub/gramene/release36/data/qtl/Release 36, January 26, 2013) [54]. It included 8,216 QTLs on 236 traits. For the 34 traits available in the panel, we excluded traits with less than 30 QTL reports, and only kept the first (Days to flower at Arkansas) from the 5 flowering time traits in our further analyses, so that 11 out of the 34 traits were finally used to validate the new approach. The numbers of SNPs from the rice diversity panel which were located in corresponding QTL regions for each trait are summarized in Table 3. Marker weights were again inferred by counting the number of publications reporting a significant QTL region as described above, and the marker weights for plant height and panicle length were plotted in Figure S3. The annotation information for these SNPs and their marker weights are provided in Table S3.

### Genomic Prediction with BLUP (Best Linear Unbiased Prediction)

The statistical model for the genomic BLUP approach is
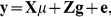
(Model 1)in which **y** is a vector of phenotypic values; *μ* is the overall mean; **g** is a multivariate normally distributed vector of genetic values for all individuals in the model; 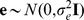 is the residual term; **X** and **Z** are incidence matrices relating the overall mean and the genetic values to the phenotypic record. We assume 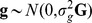 in the GBLUP approach and 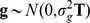 in BLUP|GA, respectively, where **T** is the matrix from equation (3) and the “GA” stands for “genetic architecture”. For TABLUP, the **TA** matrix were built according to equation (2) that proposed by Zhang *et al.* [51]. Estimated genetic values were obtained by solving the mixed model equations [73], [74] corresponding to Model 1, which are given by



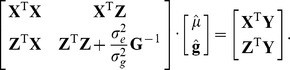



A combined AI-EM restricted maximum likelihood algorithm (AI-average information, EM-expectation maximization) was used to estimate the variance components of the model via the DMU software package [75] from the complete data and these variance components were used in the cross-validations later on.

### Genomic Prediction with BayesB

The model for BayesB [4] is given by
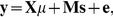
(Model 2)where **y**, **X**, *μ*, **M** and **e** are as defined in Model 1 and **s** is a vector of normally distributed and independent SNP effects. The variance of the *i*th marker effect,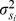 was assumed a priori to be 0 with probability of π or to follow a scaled inverse chi-squared distribution with probability of (1 – π) [4]. In our research, we chose 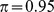 for all scenarios such that on average 5% markers were contributing to the additive genetic variance in each cycle. The MCMC chain was run for 10,000 cycles with 100 cycles of Metropolis-Hastings sampling in each Gibbs sampling, and the first 2,000 cycles were discarded as burn-in. All the samples of marker effects from later cycles were averaged to obtain the estimates of marker effects. For more details on the BayesB approach we refer to the original article [4].

### Cross-validation

A five-fold cross-validation (CV) procedure [76] was used to assess the predictive ability of the different prediction methods. In each replicate of a five-fold CV, individuals were randomly divided into five groups (folds) with equal size (in case the population size was not divisible by five, some groups included slightly more individuals than the other groups). The genetic values of all individuals in each of the five folds were predicted using records of the other four folds. Hence, in each replicate, we performed genomic prediction five times. Each individual therefore belonged once to the validation set and four times to the training set. For all scenarios, the five-fold CV was replicated 20 times, resulting in 20 average accuracies.

### Accuracy and unbiasedness

Both accuracy and predictive ability in this study were defined as the Pearson correlation coefficient between observed phenotypic values (PHE) and predicted genetic values (PGV): 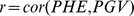. For the dairy cattle data set, the mean reliabilities for the EBVs, which were treated as phenotypes in our genomic prediction model, are 0.97, 0.97 and 0.94 for FP, MY, and SCS, respectively (Table 1). The reported results for dairy cattle can therefore be a good indicator of “accuracy” defined as the correlation between true breeding values (TBV) and genomic estimated breeding values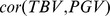. The unbiasedness was calculated as the regression coefficient of PHE on PGV, 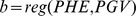. For the scenarios with low heritability traits in the dairy cattle data set, we used the original phenotypes (EBVs) rather than the simulated new phenotypes to validate different methods.

## Supporting Information

Figure S1 **Computing times for GBLUP, BLUP|GA and BayesB.** Computing times for GBLUP, BLUP|GA and BayesB (10,000 iterations) for population size *N* = 2,000 and *m* = 42,551 markers on an Intel Core i5-3470 CPU 3.2 GHz×4 with 16 GB RAM. For GBLUP and BLUP|GA, the computing time includes building the **G** matrix and solving the mixed model equations. For BayesB, the average time demanding for 10,000 iterations is shown.
(TIF)

Figure S2 **Estimated marker effects for milk yield in dairy cattle.** Estimated marker effects obtained with different population sizes (*N*). Dark blue dots represent the top 1% SNPs with the largest estimated marker effects.
(TIF)

Figure S3 **Distribution of reported QTLs positions and marker weights obtained from rice QTL list.** Reported QTLs associated with plant height (red), and panicle lenght (blue) retrieved from Gramene database (ftp://ftp.gramene.org/pub/gramene/release36/data/qtl/Release 36, January 26, 2013) [54]. Marker weights were calculated as the number of times that each marker was reported to be within a significant QTL region (QTL counts). The colored bar under each plot shows the distribution of QTL positions across the whole genome for the three traits with color keys defined in the first plot (top-right).
(TIF)

Table S1 **Accuracy and unbiasedness for traits with low heritability and small population sizes (based on the dairy cattle data set).** The best result in each block is printed in boldface.
(DOC)

Table S2 **SNP lists for the dairy cattle data set.** This table (excel format) includes the name, chromosome, physical position, trait associated, number of QTL reports, and other important information for each SNP used in this study.
(XLS)

Table S3 **SNP lists for rice diversity panel.** This table (excel format) includes the name, chromosome, physical position, trait associated, QTL report, and other information for each SNP used in this study.
(RAR)
