# Supplementary figures and images for: Improving the Accuracy of Whole Genome Prediction for Complex Traits Using the Results of Genome Wide Association Studies
Source: PLoS One. 2014 Mar 24;9(3):e93017. doi: 10.1371/journal.pone.0093017 (PMC3963961; doi:10.1371/journal.pone.0093017)

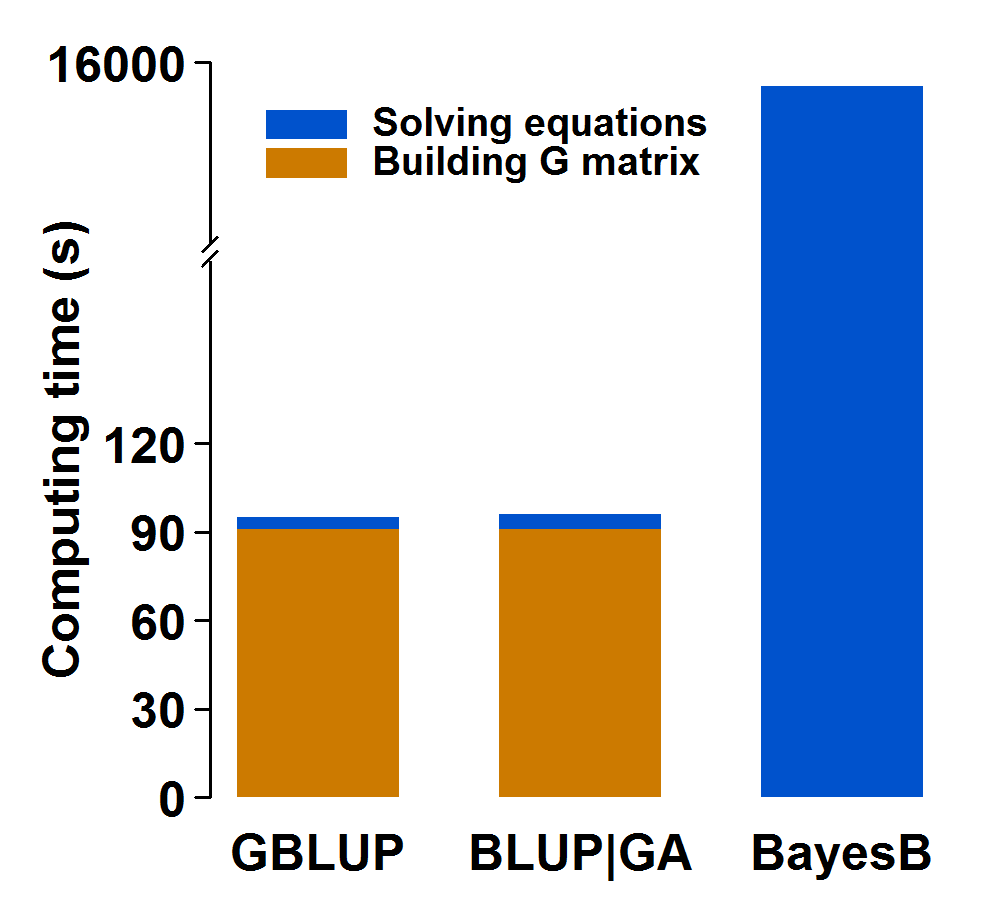

Supplement: Figure S1 — Computing times for GBLUP, BLUP|GA and BayesB. Computing times for GBLUP, BLUP|GA and BayesB (10,000 iterations) for population size N = 2,000 and m = 42,551 markers on an Intel Core i5-3470 CPU 3.2 GHz×4 with 16 GB RAM. For GBLUP and BLUP|GA, the computing time includes building the G matrix and solving the mixed model equations. For BayesB, the average time demanding for 10,000 iterations is shown. (TIF) [file pone.0093017.s001.tif]

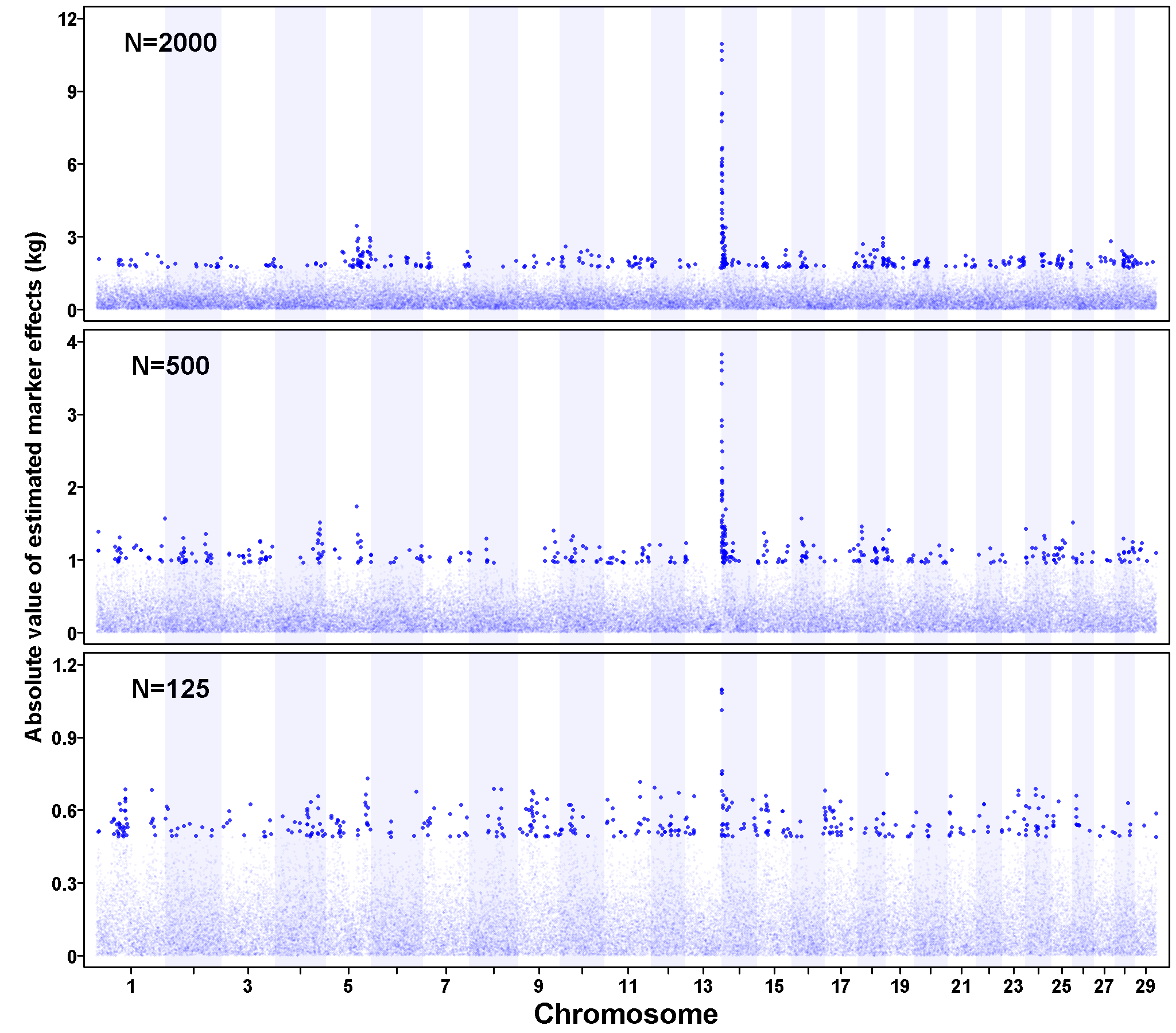

Supplement: Figure S2 — Estimated marker effects for milk yield in dairy cattle. Estimated marker effects obtained with different population sizes (N). Dark blue dots represent the top 1% SNPs with the largest estimated marker effects. (TIF) [file pone.0093017.s002.tif]

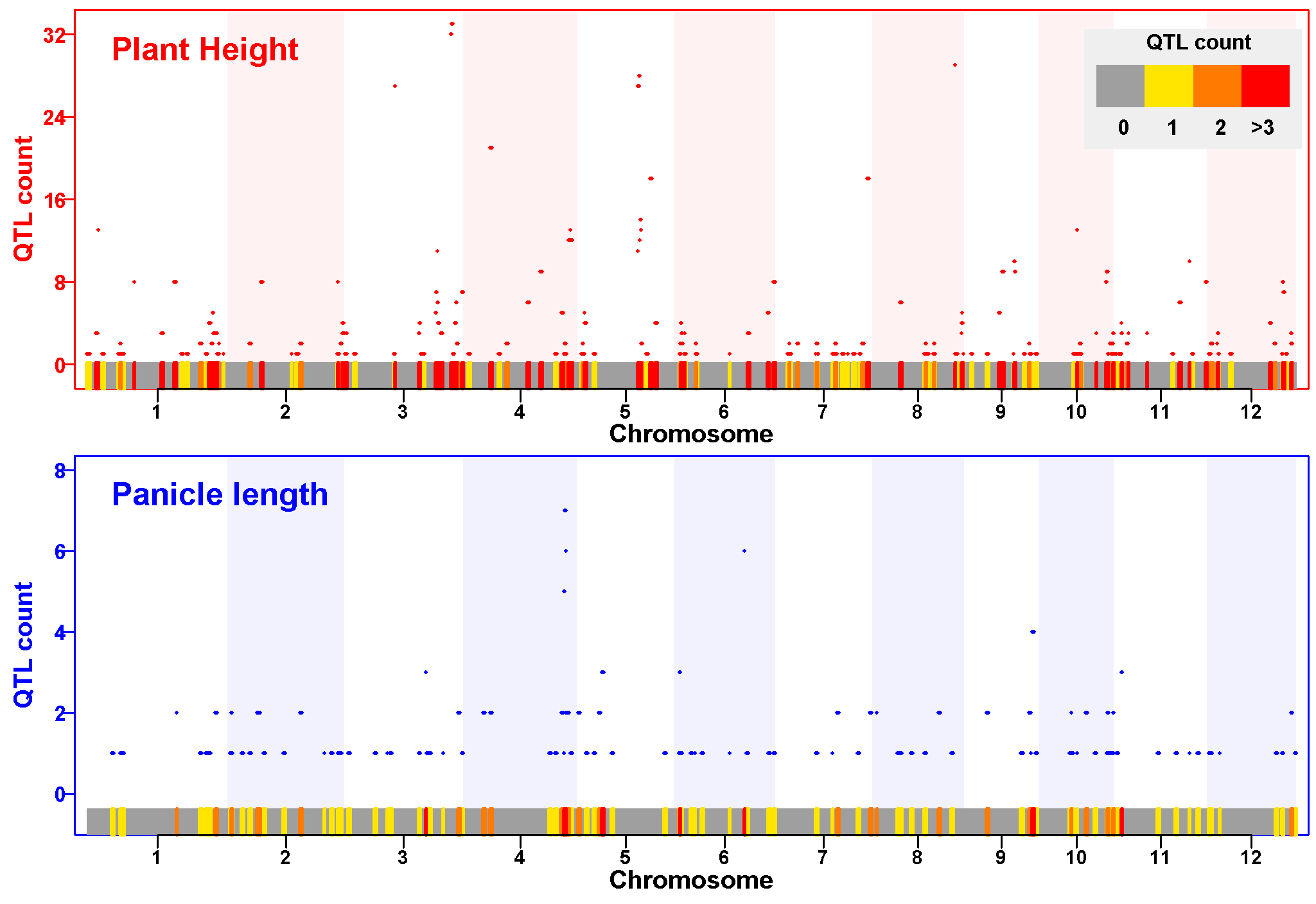

Supplement: Figure S3 — Distribution of reported QTLs positions and marker weights obtained from rice QTL list. Reported QTLs associated with plant height (red), and panicle lenght (blue) retrieved from Gramene database (ftp://ftp.gramene.org/pub/gramene/release36/data/qtl/Release 36, January 26, 2013) [54]. Marker weights were calculated as the number of times that each marker was reported to be within a significant QTL region (QTL counts). The colored bar under each plot shows the distribution of QTL positions across the whole genome for the three traits with color keys defined in the first plot (top-right). (TIF) [file pone.0093017.s003.tif]
