# Supplementary material for: Improving the Accuracy of Whole Genome Prediction for Complex Traits Using the Results of Genome Wide Association Studies
Source: PLoS One. 2014 Mar 24;9(3):e93017. doi: 10.1371/journal.pone.0093017 (PMC3963961; doi:10.1371/journal.pone.0093017)
Supplement: Table S1 — Accuracy and unbiasedness for traits with low heritability and small population sizes (based on the dairy cattle data set). The best result in each block is printed in boldface. (DOC) [file pone.0093017.s004.doc]

Supporting Table S1

| Statistics | *N* | Method | Heritability | | | |
| --- | --- | --- | --- | --- | --- | --- |
|  |  |  | Original | 0.5 | 0.3 | 0.1 |
| *r*_(EBV, GEBV)_ | 2000 | BLUP|GA | **0.751**±0.001 | 0.684±0.002 | 0.633±0.002 | **0.531**±0.005 |
|  |  | BayesB | 0.749±0.001 | **0.685**±0.001 | **0.637**±0.002 | 0.502±0.009 |
|  |  | GBLUP | 0.720±0.001 | 0.641±0.002 | 0.581±0.002 | 0.456±0.006 |
|  | 500 | BLUP|GA | 0.666±0.003 | 0.603±0.004 | **0.534**±0.004 | **0.392**±0.015 |
|  |  | BayesB | **0.670**±0.002 | **0.604**±0.004 | 0.521±0.009 | 0.289±0.017 |
|  |  | GBLUP | 0.593±0.004 | 0.513±0.004 | 0.462±0.005 | 0.292±0.018 |
|  | 125 | BLUP|GA | **0.498**±0.007 | **0.437**±0.009 | **0.360**±0.019 | **0.270**±0.025 |
|  |  | BayesB | 0.450±0.009 | 0.359±0.009 | 0.293±0.016 | 0.157±0.023 |
|  |  | GBLUP | 0.432±0.010 | 0.354±0.010 | 0.290±0.016 | 0.159±0.022 |
| *b*_(EBV,GEBV)_ | 2000 | BLUP|GA | **1.025**±0.002 | **1.007**±0.005 | **1.043**±0.009 | **1.212**±0.043 |
|  |  | BayesB | 1.027±0.002 | 1.045±0.006 | 1.123±0.012 | 1.844±0.107 |
|  |  | GBLUP | 1.042±0.002 | 1.027±0.005 | 1.088±0.010 | 1.293±0.055 |
|  | 500 | BLUP|GA | 1.152±0.005 | **1.136**±0.014 | **1.129**±0.028 | **1.334**±0.076 |
|  |  | BayesB | **1.137**±0.006 | 1.245±0.030 | 1.656±0.068 | 2.689±0.216 |
|  |  | GBLUP | 1.169±0.007 | 1.154±0.015 | 1.221±0.034 | 1.363±0.101 |
|  | 125 | BLUP|GA | **1.203**±0.020 | **1.283**±0.048 | **1.378**±0.071 | 1.449±0.192 |
|  |  | BayesB | 1.774±0.039 | 1.976±0.089 | 2.484±0.158 | 2.867±0.445 |
|  |  | GBLUP | 1.379±0.034 | 1.335±0.053 | 1.434±0.083 | **1.315**±0.181 |
